# Multidisciplinary Strategies for Tailored Anesthesia Management in Children Undergoing Radiotherapy

**DOI:** 10.3390/children13050587

**Published:** 2026-04-23

**Authors:** Salvatore Palmese, Renato Gammaldi, Alessandro Vittori, Marco Cascella

**Affiliations:** 1 Department of Anesthesia and Intensive Care, AOU “San Giovanni di Dio e Ruggi d’Aragona”, 84131 Salerno, Italy; 2 Department of Anesthesia, Critical Care and Pain Medicine, ARCO, Ospedale Pediatrico Bambino Gesù IRCCS, Piazza S. Onofrio 4, 00165 Rome, Italy; 3 Department of Medicine, Surgery and Dentistry “Scuola Medica Salernitana”, University of Salerno, 84081 Salerno, Italy

**Keywords:** pediatric radiotherapy, anesthesia, deep sedation, non-operating room anesthesia (NORA), propofol, midazolam, multidisciplinary care

## Abstract

Although radiotherapy is a cornerstone in the management of several pediatric malignancies, its administration in children poses unique anesthetic challenges. Unlike adults, pediatric patients, particularly younger children, often require repeated sedation or general anesthesia to ensure immobility and reduce psychological distress during daily treatment sessions that may extend over several weeks. This narrative review summarizes current evidence on anesthetic strategies for children undergoing radiotherapy, focusing on clinical indications, pharmacological approaches, safety considerations, and organizational aspects. We discuss the main sedation and anesthesia techniques used in non-operating room anesthesia (NORA) settings, including deep sedation with midazolam, propofol, ketamine, and dexmedetomidine, as well as general anesthesia with laryngeal mask airway management. Particular attention is given to the cumulative effects of repeated anesthetic exposure, airway management challenges in remote radiation environments, and the risk of respiratory and hemodynamic complications. The review also highlights the importance of individualized, protocol-driven management, rapid recovery strategies, and continuous remote monitoring systems. Non-pharmacological interventions and audiovisual-assisted techniques are also discussed as potential strategies to reduce anesthesia requirements in selected patients. A multidisciplinary approach involving anesthesiologists, radiation oncologists, nurses, psychologists, and technical staff is essential to optimize safety, treatment adherence, and overall quality of care. Tailored anesthetic management, supported by standardized protocols and specialized pediatric expertise, remains crucial to balancing procedural efficacy with short- and long-term safety in this vulnerable population.

## 1. Introduction

Radiotherapy is an essential part of the treatment of several pediatric malignancies, including primary central nervous system (CNS) tumors (28%), retinoblastoma (26%), neuroblastoma (18%), acute leukemia (9%), Wilms tumor (5%), rhabdomyosarcoma (7%), Langerhans cell histiocytosis (4%), and other malignancies (3%), according to epidemiological registry data [1]. In adult patients, radiotherapy administration is generally easier due to their ability to actively cooperate, reducing the need for anesthesia in special cases such as those with cognitive disability, movement disorders, or claustrophobia. In contrast, radiotherapy in children presents unique challenges. Pediatric patients, especially the youngest, are unable to remain still during treatment sessions and often experience high levels of anxiety and fear. For this reason, anesthesia becomes essential not only to ensure immobility, but also to reduce the psychological trauma associated with treatment. Notably, radiotherapy cycles in children can include 15 to over 30 sessions, thus requiring daily sedation or anesthesia for several consecutive weeks. This frequency introduces additional challenges, such as managing cumulative side effects and the need for rapid recovery between sessions [2]. Furthermore, radiotherapy techniques vary significantly and include photon beam therapy, proton beam therapy, and, less frequently in children, brachytherapy. Proton therapy, characterized by the Bragg peak phenomenon, allows for highly localized dose deposition with reduced exit radiation, while photon therapy involves a broader dose distribution. Moreover, proton beam therapy may require longer setup times and stricter immobilization precision compared to conventional photon therapy. In addition, treatment rooms are often larger, and access to the patient may be further restricted due to gantry configuration. These factors may increase anesthetic complexity, particularly in very young children or in craniospinal irradiation. Collectively, these technical differences may influence treatment duration, immobilization requirements, and anesthetic planning, particularly in complex cranial or head-and-neck tumors.

Despite the widespread use of sedation and general anesthesia in pediatric radiotherapy, clinical practices remain highly heterogeneous across institutions, with significant variability in anesthetic agents, airway management strategies, monitoring standards, and staffing models. This lack of standardization highlights the urgent need for harmonized, evidence-informed clinical pathways [3].

This article was designed as a narrative review with a focused literature search intended to identify clinically relevant publications on anesthesia management in pediatric radiotherapy. The aim of this review is to combine the available scientific evidence with the authors’ clinical experience. The interest for pediatricians and anesthetists is to have guidance in patient selection and practical management during radiotherapy sessions.

## 2. Search Strategy and Study Selection

This narrative review was conducted in accordance with the SANRA (Scale for the Assessment of Narrative Review Articles) recommendations to improve methodological transparency and scientific rigor [4]. The search was performed in PubMed, Scopus, and Web of Science using combinations of terms related to pediatric radiotherapy, anesthesia, sedation, non-operating room anesthesia, and airway management. Although priority was given to English-language studies published in the last 15 years, older landmark papers and relevant guidelines were included when considered important for historical or clinical context. Studies were considered relevant if they addressed anesthetic or sedation strategies in pediatric radiotherapy settings, including clinical studies, reviews, and guidelines, while publications not directly related to the topic were excluded. Study selection was based on screening of titles and abstracts, followed by full-text evaluation for clinical relevance. Greater emphasis was placed on multicenter studies, systematic reviews, and high-volume institutional experiences when available. A supplementary search was performed by screening the reference lists of the selected papers to identify additional relevant studies. Further literature was identified based on the authors’ expertise.

Given the narrative purpose of the review and the heterogeneity of the available literature, the retrieved evidence was synthesized to provide a clinically oriented overview rather than a formal systematic appraisal.

## 3. Clinical Context and Operative Environment

Anesthesia in pediatric radiotherapy is essential to ensure that treatments are performed safely and with maximum effectiveness. Radiotherapy frequently requires preliminary simulation sessions, including CT or MRI imaging, which may be performed under the same anesthetic episode as treatment planning. Safe transfer between imaging suites and the radiotherapy bunker must therefore be considered during anesthetic planning. Immobilization devices such as thermoplastic face masks or body molds are often used to ensure treatment precision and may further complicate airway access and patient positioning.

Children, especially the youngest, are often unable to remain still during sessions [5]. Moreover, unlike adults, pediatric patients have unique characteristics that must be carefully considered. For example, anxiety levels are often very high, their ability to cooperate is limited, and they are more exposed to the side effects of radiotherapy and anesthesia [6]. Separation from parents and the use of complex equipment accentuate this discomfort, requiring targeted anesthesia strategies to prevent further trauma [7]. Furthermore, age is a crucial factor. Younger patients usually require general anesthesia to ensure immobility, especially in advanced treatments such as proton therapy [8]. In older and more cooperative patients, deep or conscious sedation techniques may reduce the need for general anesthesia, minimizing risks and promoting faster recovery. However, they require careful selection of candidates [9].

Anesthesia in a radiotherapy bunker presents unique challenges, requiring a specialized approach due to suboptimal conditions and remote monitoring [10]. Additionally, the lack of a specialized pediatric anesthesia team in some facilities increases the risk of complications [11,12,13]. This NORA (Non-Operating Room Anesthesia) approach requires a highly specialized approach. Unlike conventional operating rooms, radiotherapy occurs in environments specifically designed to protect against radiation, but not to perform anesthetic procedures. This means that the anesthesia team often finds themselves working in suboptimal conditions, with limited access to the patient and the need to monitor the patient remotely via cameras and other remote surveillance systems [10].

Another significant issue is that not all pediatric hospitals have a radiotherapy service, as pediatric oncology cases are relatively few. As a result, radiotherapy sessions often take place in general facilities, where a team specialized in pediatric anesthesia may be lacking. This situation increases the risk of complications, especially due to the possible lack of specific experience of the staff in the management of anesthesia in children, especially in such a unique environment as a radiotherapy bunker. Importantly, evidence suggests that centers with higher volumes of pediatric anesthetic procedures tend to report lower complication rates, likely reflecting the impact of dedicated pediatric anesthesia teams and institutional experience. Therefore, expertise concentration and structured training programs may significantly influence safety outcomes in this highly specialized setting [2].

## 4. Physiological and Psychological Aspects

Anesthesia in pediatric patients requires a personalized approach to manage the physiological and psychological specificities of children. Age-related physiological variability significantly influences pharmacokinetics and pharmacodynamics. Airway management is critical, especially during radiotherapy, due to the small size and greater vulnerability to obstruction. The use of a laryngeal mask airway (LMA) is preferred compared to open airways, as it allows for safer management in settings with limited patient access [14,15,16]. LMA may represent a good compromise for the management of patients who present difficult management with a face mask, or when tracheal intubation is potentially difficult (such as in patients with facial tumors), in a NORA setting. In fact, in cases of head and neck cancer, the necessary immobilization can further complicate airway patency, making the use of supraglottic devices and careful management essential to avoid respiratory complications. However, endotracheal intubation should be used whenever it is necessary to protect the airway, for example, when the patient has numerous secretions capable of irritating the airway [17,18].

Prolonged positioning on rigid treatment tables increases the risk of pressure-related injuries, particularly in malnourished or chronically ill children. Additionally, partial undressing required for laser alignment and target visualization may predispose to peri-procedural hypothermia. Active warming strategies applied outside the radiation field and careful padding of pressure points are therefore recommended.

From a psychological point of view, children often experience intense anxiety, influenced by factors such as separation from parents and the strangeness of the medical environment. Non-pharmacological interventions, such as audiovisual devices, interactive games, and the presence of family members, can reduce anxiety and improve cooperation, limiting the need for general anesthesia. In more complex cases, a multidisciplinary approach involving anesthesiologists and psychologists is crucial to emotionally support the child and parents, thus improving long-term outcomes. In addition, desensitization sessions can psychologically prepare the child, reducing anxiety and promoting better acceptance of treatment [19].

## 5. Indications and Techniques

Anesthetic management in children undergoing radiotherapy must consider the specific clinical needs of the patient and the frequency of sessions, requiring a personalized approach. Radiotherapy cycle can include 15 to over 30 sessions, which means that children must be sedated or anesthetized daily for a prolonged period, often for several consecutive weeks. This repetitiveness introduces additional challenges in anesthetic management, as continuous exposure to anesthetic drugs and the need for repeated inductions represent an additional risk for both the immediate safety and long-term well-being of the patient [20].

The radiotherapy procedure generally lasts between 5 and 15 min, depending on the number of sites to be irradiated. Given the short duration of the sessions, sedation is often preferred over general anesthesia, which remains indicated especially for the simulation process or in specific cases such as very young patients or those with special clinical needs [10]. Nevertheless, significant disparities exist between high-income and resource-limited settings. In some low- and middle-income countries, anesthetic management may rely more frequently on intramuscular ketamine due to the limited availability of intravenous agents, infusion pumps, monitoring equipment, or trained pediatric anesthesiologists [2]. These contextual differences may influence safety profiles and highlight the importance of adaptable, resource-sensitive clinical frameworks.

### 5.1. Deep Sedation

Deep sedation can be defined as a transition from conscious sedation to unconsciousness, with loss of both verbal communication and protective reflexes [21]. This technique is indicated for older children, especially those over 7 years of age, who can tolerate brief periods of immobilization without complete loss of consciousness. Deep sedation allows the child to remain drowsy while maintaining an open airway without the need for intubation [22]. Medications commonly used for deep sedation include:

- Midazolam: A benzodiazepine hypnotic/sedative that works by stimulating gamma-aminobutyric acid (GABA)A receptors. Midazolam is the most used drug for pediatric sedation [23]. Midazolam is highly protein-bound to albumin, requiring dose adjustment in hypoalbuminemic patients, and is metabolized to inactive metabolites.
- Propofol: A short-acting agent used for the induction and maintenance of anesthesia; propofol is particularly suitable for short procedures such as radiotherapy sessions due to its favorable pharmacokinetic profile [11]. It works by stimulating GABAA receptors and activating chloride channels, inhibiting synaptic transmission. Propofol may reduce cardiac output and attenuate the compensatory response, causing bradycardia [24]. The main disadvantage is the risk of apnea [25].
- Ketamine: A phencyclidine derivative that acts on N-methyl-D-aspartate (NMDA) receptors to produce dissociative anesthesia [26]. Although ketamine may increase secretions and cause agitation, it is beneficial in some patients because of its capacity to promote bronchodilation, preserving pharyngeal and laryngeal reflexes. However, it should be avoided in patients with seizure disorders and increased intracranial pressure. For procedural sedation, it is also combined with midazolam [23].
- Dexmedetomidine: An alpha-2 agonist increasingly used for pediatric sedation. It acts on alpha-2 receptors located in the periphery, brain (locus coeruleus), and spinal cord, producing sedation and antinociception. In the setting of fractionated radiotherapy, intranasal dexmedetomidine appears to achieve more consistent and satisfactory sedation than oral ketamine combined with midazolam [23].
- Opioids, although effective for painful procedures, are rarely used during radiotherapy due to their respiratory depressant effects and are reserved for special cases [27] (Table 1).

### 5.2. General Anesthesia

General anesthesia is a medically induced, reversible state of total unconsciousness and pain relief [28].

General anesthesia is mostly indicated in younger children (under 4 years) or in those with special clinical needs, such as behavioral or neurological disorders, who require complete immobilization [3]. In these cases, airway management is usually performed with an LMA, reducing the risk of complications related to endotracheal intubation. However, general anesthesia can carry a higher risk of respiratory and cardiovascular complications, as well as requiring a longer recovery period and more rigorous postoperative monitoring [29]. Nevertheless, evidence from high-volume centers suggests that when performed by dedicated pediatric anesthesia teams using standardized protocols, general anesthesia with LMA can be associated with low complication rates and rapid turnover times, even across prolonged treatment cycles [30,31].

### 5.3. Conscious Sedation

Conscious sedation is a technique whereby patients undergo a drug-induced depression of their consciousness but retain the ability to self-ventilate, maintain protective reflexes, and respond to verbal or light-pressure stimuli [32]. This technique, although less frequently used, is indicated in selected patients, especially for older children, who can tolerate the procedure without the need for deep immobilization. Conscious sedation significantly reduces recovery times and complications but is not suitable for younger patients or those with poor sedation tolerance [33].

In conclusion, the choice of anesthetic agent and administration technique should be individualized based on the patient’s clinical condition, tumor type, and individual response to drugs. An evidence-based protocol and rigorous monitoring are essential to ensure safe and effective treatment throughout the entire radiotherapy cycle [34].

A comparative overview of deep sedation and general anesthesia in pediatric radiotherapy is provided in Table 2. For patients aged between 4 and 7 years, the choice between deep sedation and general anesthesia depends on the patient’s conditions (cognitive status, weight), the organization of the individual center, and the experience of the anesthesiologist [35].

## 6. Anesthesia Management

A typical anesthesia management for pediatric radiotherapy involves a thorough pre-procedural assessment to identify any specific risks. Patients are usually fasted according to current ASA fasting guidelines to reduce the risk of aspiration. Premedication may include oral, intranasal, or intravenous midazolam to reduce anxiety. During the procedure, propofol is used for the induction of anesthesia, while maintenance may be performed with either continuous propofol infusion or halogenated gases, such as sevoflurane, which is commonly used in children due to its rapid onset and low blood solubility. The use of adjunctive agents, such as ketamine or dexmedetomidine, may be considered depending on the patient’s needs. LMA management is often preferred to reduce complications.

Traditionally, intravenous or combined (intravenous and inhalation) anesthesia has been the standard of care for these cases. However, recent studies, such as that conducted by García-Aroca et al. [36], suggest that inhalation anesthesia, particularly the use of sevoflurane administered via LMA, is a valid and safe option. In our recent cohort experience involving 420 anesthetic sessions in pediatric radiotherapy, the implementation of a standardized LMA-based protocol was associated with a low overall complication rate (1.9%), rapid recovery times, and no major adverse events [22,30]. Specifically, the protocol provided the use of short-acting agents (intravenous propofol for induction and sevoflurane for maintenance) and airway management with an LMA, and ventilation was maintained in pressure-support mode with continuous capnography [30,37].

Regardless of the technique implemented, the patient should be visually monitored in the radiation treatment room using two cameras, one focused on the patient so that chest movement (breathing) can be observed, and the other on the portable monitor. The cameras can be repositioned via remote control to allow continuous monitoring as the radiation treatment progresses. This approach can provide an optimal balance of safety, efficacy, and comfort for the pediatric patient, while minimizing the risks associated with repeated exposure to anesthesia during radiation therapy. At the end of the session, patients require monitoring until they are fully awake and hemodynamically stable before being discharged from the recovery room [38].

A careful and effective periprocedural protocol must address other critical aspects. In radiotherapy bunkers, the anesthetic machine is often positioned at a distance from the patient to avoid radiation exposure. Extended breathing circuits, long intravenous lines, and remote monitoring systems are therefore required. These technical adaptations may increase dead space, delay drug administration feedback, and complicate emergency airway access, necessitating meticulous preparation and simulation-based team training.

## 7. Potential Complications and Management

Anesthesia in pediatric radiation therapy carries the risk of several complications [39]. However, a major limitation of the available literature is the lack of standardized definitions for anesthesia-related complications. Variability in reporting criteria makes it difficult to compare outcomes across centers and may underestimate the true incidence of adverse events [2].

Respiratory complications are among the most common and may include apnea, oxygen desaturation, airway obstruction, and, in rare cases, bronchospasm and laryngospasm. In our cohort analysis, laryngospasm represented the most frequent respiratory event, yet its incidence remained below 1%, with no cases requiring tracheal intubation or ICU admission [30,40]. Many children undergoing radiotherapy receive concurrent chemotherapy and opioids, which may delay gastric emptying and increase the risk of aspiration. Chemotherapy-induced mucositis may predispose to airway irritation and laryngospasm, particularly in cases of repeated airway instrumentation. These factors must be considered during airway device selection and fasting assessment. The management of these events is critical, as the patient is often in a remote environment and monitored remotely via cameras.

Hemodynamic instability may manifest as bradycardia, tachycardia, hypotension, or hypertension and can result from multiple interacting factors, including anesthetic drug effects, hypovolemia, underlying cardiac dysfunction, pain or inadequate sedation, and autonomic responses to airway manipulation. Propofol-related vasodilation may precipitate hypotension [11], whereas agents such as ketamine may induce transient increases in heart rate and blood pressure due to sympathetic stimulation [26]. Repeated anesthetic exposure, concomitant chemotherapy, dehydration, and electrolyte imbalances may further exacerbate cardiovascular lability. Continuous monitoring and prompt titration of anesthetic agents, along with appropriate fluid management and readiness to administer vasoactive support, are essential to maintain hemodynamic stability in this vulnerable population.

In addition, the use of immunosuppressive drugs, such as chemotherapy and corticosteroids, increases the susceptibility of children to the development of upper respiratory tract infections (URTI). These infections can further complicate anesthesia management and ventilation during radiotherapy.

The presence of URTI can make the administration of general anesthesia risky, but interrupting the radiotherapy cycle could have negative consequences on the oncological treatment. For this reason, the decision to interrupt or continue treatment must be carefully evaluated, balancing the risks associated with the respiratory infection with those resulting from an interruption of the radiotherapy cycle. In some cases, it may be necessary to adapt the anesthesia protocol to minimize the risks, for example, by using less invasive anesthesia techniques or more rigorous monitoring.

Postoperative nausea and vomiting, although less common, as most children are already on antiemetic therapy, can still negatively affect recovery and increase patient discomfort. In addition, the need to repeat anesthesia for several radiotherapy sessions (which can last up to six consecutive weeks) exposes children to cumulative risks of adverse effects, including both immediate complications and potential long-term effects on development [41].

Prevention and effective management of these complications requires a multidisciplinary approach and the adoption of specific strategies. The use of balanced anesthesia, combining propofol with adjuvants such as midazolam or ketamine, is recommended to reduce the risk of respiratory and cardiovascular complications. It is essential to carefully monitor the patient’s vital signs using advanced tools, including continuous pulse oximetry, electrocardiography, and blood pressure monitoring at regular intervals.

Furthermore, rapid recovery techniques, such as precise titration of propofol and limitation of opioid use, are essential to minimize the risk of respiratory depression and to facilitate a more rapid recovery, thus reducing the time spent in the recovery room. The use of supportive strategies, such as fluid preparation to prevent hypotension and immediate availability of rescue medications, can further improve the management of complications [42]. Finally, regular monitoring of laboratory parameters, including hemoglobin levels, platelet counts, and renal function, is essential during prolonged radiotherapy cycles. Minimum acceptable thresholds should be agreed upon with the oncology team to ensure both anesthetic safety and oncologic treatment efficacy [43].

## 8. Organizational Aspects and Interdisciplinary Collaboration

In pediatric radiotherapy, anesthesia management requires close collaboration between anesthesiologists, oncologists, radiation oncologists, nurses, and technicians to ensure the safety and effectiveness of the treatment. Anesthesiologists must customize the protocol according to the specific needs of the patient, in collaboration with the team, while nurses and technicians monitor and prepare the patient, intervening promptly in case of complications. For example, thermoplastic face masks used for head and neck immobilization may interfere with airway patency, restrict chest wall movement, and delay immediate access to the airway in emergencies. Careful airway device positioning and contingency planning are essential when such immobilization systems are in place.

In this complex scenario, interdisciplinary collaboration is crucial in repeated sedations, allowing to adapt therapeutic strategies in response to clinical changes, reducing the risk of complications and improving treatment adherence. Optimizing the workflow and adopting standardized protocols is essential to maintain efficiency and reduce risks [44]. Radiation safety considerations also extend to anesthesia personnel. Although staff members exit the bunker during irradiation, workflow planning must ensure the safe positioning of equipment and minimize repeated exposure during patient setup phases. Therefore, through interdisciplinary collaboration, constant patient monitoring remains essential to promptly identify and manage any complications, improving the overall quality of the service offered.

## 9. Ethical and Legal Considerations

Informed consent is crucial in pediatric treatment, especially for invasive procedures such as radiotherapy and anesthesia. Parents must receive clear information about the risks, benefits, and possible complications of anesthesia, including therapeutic alternatives and the consequences of refusing treatment. This process must be carefully documented, considering specific risks such as the use of drugs in patients with pre-existing conditions or the increased risk of infections due to immunosuppression [45].

Anesthesiologists and the medical team must ensure that procedures comply with current regulations and accepted standards of practice, with ongoing monitoring and appropriate management of complications. If the anesthesia-related risk is too high, such as in the case of severe respiratory failure or hemodynamic instability, it may be necessary to postpone or discontinue radiotherapy. This decision must be carefully discussed with the family, considering the risks of continuing or interrupting treatment [46]. In case of clinical deterioration during treatment, it is essential to communicate clearly with the family to make shared decisions in the best interest of the child.

These ethical considerations become even more complex when radiotherapy is delivered with palliative intent. In this setting, the primary objective shifts from disease control to symptom relief and quality-of-life preservation. Consequently, anesthetic management must be carefully aligned with the overall goals of care. In the context of palliative radiotherapy, the evaluation of anesthesia should extend beyond procedural safety to include proportionality of intervention, expected clinical benefit, and potential burden for the child and family. A more conservative and comfort-oriented approach is often appropriate, prioritizing symptom control while minimizing additional stress or invasive measures. Decisions to continue, modify, or discontinue radiotherapy must adhere to the ethical principle of non-maleficence, ensuring that interventions meaningfully contribute to the child’s well-being. In some cases, this may justify limiting or suspending aggressive treatments in favor of a palliative care pathway centered on dignity, comfort, and family support.

## 10. Conclusions

Anesthesia for pediatric radiotherapy represents a highly specialized field that requires the integration of clinical expertise, organizational efficiency, and psychological sensitivity. The need for repeated sedation or general anesthesia over prolonged treatment cycles exposes children to cumulative risks, making individualized, protocol-driven management essential. Deep sedation with short-acting agents such as propofol, often combined with adjuncts including midazolam, ketamine, or dexmedetomidine, has emerged as an effective strategy for many patients, while general anesthesia remains necessary in younger or non-cooperative children. Careful airway management, continuous remote monitoring, and rapid recovery protocols are critical components of safe practice in the NORA setting.

Beyond pharmacological strategies, non-pharmacological interventions and audiovisual-assisted techniques offer promising opportunities to reduce anesthesia exposure in selected patients, highlighting the importance of psychological preparation and family-centered care. Ultimately, optimal outcomes depend on a multidisciplinary model that integrates anesthesiologists, radiation oncologists, pediatric oncologists, nurses, psychologists, and technical staff. Standardized protocols, specialized pediatric expertise, and continuous quality assessment are key elements in minimizing complications and improving treatment adherence.

Unfortunately, as is often the case in pediatrics, the literature base is largely retrospective, heterogeneous, and center-dependent.

Given the current gaps in the literature, future research should focus on long-term neurodevelopmental outcomes associated with repeated anesthetic exposure, comparative effectiveness studies between sedation strategies, and the development of structured clinical pathways tailored to different age groups and oncologic settings. Strengthening evidence-based practice in this area will be crucial to ensuring both procedural efficacy and long-term safety for this vulnerable population.

## Figures and Tables

**Table 1:** Main anesthetic agents used in pediatric radiotherapy: pharmacological characteristics, advantages, and limitations.

Agent	Mechanism of Action	Onset/Duration	Advantages	Limitations/Risks	Preferred Clinical Context
Midazolam	GABAA receptor agonist (benzodiazepine)	Onset: 30–60 s (IV) Duration: 20–80 min	Anxiolysis; amnesia; multiple routes (oral, IN, IV)	Respiratory depression (dose-dependent); paradoxical reactions; protein binding considerations	Premedication; mild-to-moderate sedation
Propofol	GABAA receptor modulation; chloride channel activation	Onset: 30–60 s Duration: 5–10 min	Rapid onset and recovery; easy titration; ideal for short procedures	Apnea (20–30%); hypotension; bradycardia; no analgesia	Deep sedation; short repetitive sessions
Ketamine	NMDA receptor antagonist	Onset: 30–60 s Duration: 10–20 min	Preserves airway reflexes; bronchodilatory effect; hemodynamic stability	Hypersalivation; emergence agitation; contraindicated in ↑ICP	Reactive airway disease; need to preserve spontaneous breathing
Dexmedetomidine	α2-adrenergic agonist (locus coeruleus)	Onset: gradual (loading over 10 min) Infusion-based	Sedation with minimal respiratory depression; analgesic-sparing	Bradycardia; hypotension; slower onset	Adjunct sedation; longer procedures
Sevoflurane (GA)	Volatile anesthetic; GABA-mediated CNS depression	Rapid induction and emergence	Non-invasive induction; useful without IV access	Airway irritation (rare); postoperative agitation	General anesthesia; simulation sessions; younger children
Balanced anesthesia (Propofol + adjuncts)	Multimodal approach	Agent-dependent	Reduced total drug dose; improved hemodynamic stability	Requires experienced team	Repetitive radiotherapy cycles

Abbreviations: IV, intravenous; IN, intranasal; GA, general anesthesia; NMDA, N-methyl-D-aspartate; GABA, gamma-aminobutyric acid; ICP, intracranial pressure.

**Table 2:** Comparison between deep sedation and general anesthesia for pediatric radiotherapy in non-operating room anesthesia settings.

Domain	Deep Sedation	General Anesthesia
Primary goal	Reduce anxiety and motion while preserving spontaneous ventilation when possible	Ensure complete immobility and airway control
Typical candidates	Older and more cooperative children (often ≥7 years), selected cases with adequate airway reserve	Younger children (often <4 years) and patients with limited cooperation (e.g., neurodevelopmental/behavioral disorders)
Common agents	Propofol ± adjuncts (midazolam, ketamine, dexmedetomidine)	Inhalational (e.g., sevoflurane) and/or intravenous techniques, often with supraglottic airway
Airway management	Preferably spontaneous breathing; airway support as needed; close vigilance for obstruction	Usually supraglottic airway (e.g., LMA) or endotracheal intubation in selected cases
Monitoring constraints (radiotherapy bunker)	Remote monitoring required; limited access may increase risk if airway obstruction occurs	Remote monitoring required; airway secured may improve safety in limited-access environments
Hemodynamic profile	Agent-dependent; propofol may cause hypotension/bradycardia	Agent- and technique-dependent; generally predictable but requires full anesthetic setup
Respiratory risk	Higher risk of hypoventilation/apnea with deeper propofol titration; obstruction possible	Risk of airway events exists but airway is usually controlled; emergence agitation can occur
Recovery and turnover	Typically rapid recovery; advantageous for daily short sessions	Recovery may be longer; may increase post-anesthesia care needs
Suitability for repeated daily sessions	Often preferred when feasible due to rapid recovery and streamlined workflow	Often necessary for repeated sessions in very young/non-cooperative children despite higher resource needs
Main advantages	Short onset/recovery; reduced invasiveness; may lower overall burden if well selected	Reliable immobility; controlled airway; potentially safer in children with high motion/anxiety or limited cooperation
Main limitations	Requires careful patient selection and an experienced team; risk of apnea/airway obstruction in a limited-access setting	Higher resource utilization; longer recovery; potentially increased cumulative exposure and physiological stress
When to favor	Short treatments (5–15 min), cooperative child, stable airway, goal of rapid recovery	Simulation, proton therapy/complex immobilization, very young age, poor cooperation, or high risk of movement

Abbreviation: LMA, laryngeal mask airway.

## Data Availability

No new data were created or analyzed in this study.

## References

[1] Gupta M., Garg R. (2017). Radiation Therapy for Children: Periprocedural Anaesthetic Concerns. J. Anesth. Crit. Care Open Access.

[2] Turcas A., Boterberg T., Frykholm P., Gaze M., Hendriks M., Jouglar E., Kostense Z., Maduro J., Mondardini M.C., Naik V. (2026). Anesthesia in Pediatric Radiotherapy: A Systematic Literature Review by the SIOP Europe Working Group on Pediatric Anesthesia in Radiation Therapy. Pediatr. Blood Cancer.

[3] Li L.W., Chua G.W., Wenjun K., Bong C.L. (2021). Anaesthesia for Radiotherapy in Paediatric Oncology—A Retrospective Observational Study in an Asian Population. Chin. Clin. Oncol..

[4] Baethge C., Goldbeck-Wood S., Mertens S. (2019). SANRA-A Scale for the Quality Assessment of Narrative Review Articles. Res. Integr. Peer Rev..

[5] McMullen K.P., Hanson T., Bratton J., Johnstone P.A.S. (2015). Parameters of Anesthesia/Sedation in Children Receiving Radiotherapy. Radiat. Oncol..

[6] Shimazu Y., Otsuki R., Murakami M., Konishi A., Kan K., Seto I., Yamaguchi H., Tsubokura M., Hattori H. (2020). Age as a Decisive Factor in General Anaesthesia Use in Paediatric Proton Beam Therapy. Sci. Rep..

[7] Veterini A.S., Putri H.S., Pasaribu U.P. (2022). Anesthesia Services for Children’s Radiotherapy During Pandemic COVID-19 in 2020: Experience from East Indonesia. J. Anestesiol. Indones..

[8] Yıldırım İ., Çelik A.İ., BBay S., Pasin Ö., Tütüncü, AÇ (2019). Propofol-Based Balanced Anesthesia Is Safer in Pediatric Radiotherapy. J. Oncol. Pharm. Pract..

[9] Nelson O., Bailey P.D. (2017). Pediatric Anesthesia Considerations for Interventional Radiology. Anesth. Clin..

[10] Harris E.A. (2010). Sedation and Anesthesia Options for Pediatric Patients in the Radiation Oncology Suite. Int. J. Pediatr..

[11] LaRiviere M.J., Shah Y.B., Cummings E.R., Clegg K., Doucette A., Struyk B.P., Lustig R.A., Kurtz G., Hill-Kayser C.E. (2022). General Anesthesia for Pediatric Radiation Therapy in the Era of COVID-19. Adv. Radiat. Oncol..

[12] Wolfler A., De Silvestri A., Camporesi A., Ivani G., Vittori A., Zadra N., Pasini L., Astuto M., Locatelli B., Cortegiani A. (2020). Pediatric Anesthesia Practice in Italy: A Multicenter National Prospective Observational Study Derived from the APRICOT Trial. Minerva Anestesiol..

[13] Vittori A., Tritapepe L., Chiusolo F., Rossetti E., Cascella M., Petrucci E., Pedone R., Marinangeli F., Francia E., Mascilini I. (2023). Unplanned Admissions after Day-Case Surgery in an Italian Third-Level Pediatric Hospital: A Retrospective Study. Perioper. Med..

[14] Villablanca N., Valls N., González R. (2023). Techniques and Complications of Anesthesia in Pediatric Radiotherapy: A Retrospective Cohort Study. J. Pediatr. Hematol. Oncol..

[15] Infosino A. (2002). Pediatric Upper Airway and Congenital Anomalies. Anesth. Clin. N. Am..

[16] Giraud O., Bourgain J.L., Marandas P., Billard V. (1997). Limits of Laryngeal Mask Airway in Patients after Cervical or Oral Radiotherapy. Can. J. Anaesth..

[17] Rades D., Münte S., Baumann R., Karstens J.H., Piepenbrock S., Leuwer M. (2001). Avoiding Tracheal Intubation in Children during Craniospinal Irradiation. Paediatr. Anaesth..

[18] Batista N.M., Corrigan M., Chen J.G. (2025). Procedural Sedation Performed by Pediatric Critical Care Physicians for Children Undergoing Daily Radiation Therapy Is Effective and Safe. Pediatr. Hematol. Oncol..

[19] Gutkin P.M., Donaldson S.S., Skinner L., Callejas M., Cimino J., Lore J., Bush K., Hiniker S.M. (2021). Use of Audiovisual Assisted Therapeutic Ambience in Radiotherapy (AVATAR) for Anesthesia Avoidance in a Pediatric Patient with Down Syndrome. Adv. Radiat. Oncol..

[20] Anghelescu D.L., Burgoyne L.L., Liu W., Hankins G.M., Cheng C., Beckham P.A., Shearer J., Norris A.L., Kun L.E., Bikhazi G.B. (2008). Safe Anesthesia for Radiotherapy in Pediatric Oncology: St. Jude Children’s Research Hospital Experience, 2004–2006. Int. J. Radiat. Oncol. Biol. Phys..

[21] The Administration of Moderate and Deep Sedation: Legal, Ethical Issues for Non-Anesthetist RNs—Sedation Certification. https://sedationcertification.com/administration-moderate-deep-sedation-legal-ethical-issues-non-anesthetist-rns/.

[22] Owusu-Agyemang P., Grosshans D., Arunkumar R., Rebello E., Popovich S., Zavala A., Williams C., Ruiz J., Hernandez M., Mahajan A. (2014). Non-Invasive Anesthesia for Children Undergoing Proton Radiation Therapy. Radiother. Oncol..

[23] Das R., Das R., Jena M., Janka J., Mishra S. (2022). Procedural Sedation in Children for Fractionated Radiation Treatment: Intranasal Dexmedetomidine versus Oral Midazolam and Ketamine. Indian J. Anaesth..

[24] Warpechowski P., Warpechowski R.B., De Lima B.A., Pinto E.F.A., Bastos M.L.S., Eibel B., Trindade R.D., Leiria T.L. (2025). Effects of Propofol in the Cardiac Conduction System in Electrophysiologic Study: Systematic Review and Meta-Analysis. Anesth. Res..

[25] Chidambaran V., Costandi A., D’Mello A. (2015). Propofol: A Review of Its Role in Pediatric Anesthesia and Sedation. CNS Drugs.

[26] Simonini A., Brogi E., Cascella M., Vittori A. (2022). Advantages of Ketamine in Pediatric Anesthesia. Open Med..

[27] McFadyen J.G., Pelly N., Orr R.J. (2011). Sedation and Anesthesia for the Pediatric Patient Undergoing Radiation Therapy. Curr. Opin. Anaesthesiol..

[28] Anestesia Generale—Preparazione All’Intervento Chirurgico. https://services.nhslothian.scot/preparingforsurgery/royal-infirmary-of-edinburgh/types-of-anaesthetic/general-anaesthesia/.

[29] Buchsbaum J.C., McMullen K.P., Douglas J.G., Jackson J.L., Simoneaux R.V., Hines M., Bratton J., Kerstiens J., Johnstone P.A.S. (2013). Repetitive Pediatric Anesthesia in a Non-Hospital Setting. Int. J. Radiat. Oncol. Biol. Phys..

[30] Palmese S., Secondulfo C., Caterino V., Santaniello G., Siglioccolo A., Cascella M., Gammaldi R., Bilancio G. (2025). Standardized Anesthetic Protocol in Pediatric Radiotherapy: A Retrospective Analysis of Clinical Efficacy and Outcomes. Radiother. Oncol..

[31] Zhang S., Zhao B., Chen D., Qi Y., Ma Y., Ma J., Xie W., Guo H. (2021). Anesthetic Management of Precise Radiotherapy under Apnea-like Condition. J. Int. Med. Res..

[32] Bean T., Aruede G. (2026). Conscious Sedation in Dentistry. StatPearls.

[33] Owusu-Agyemang P., Tsai J.Y., Kapoor R., Van Meter A., Tan G.M., Peters S., Opitz L., Pedrotti D., DeSoto H.S., Zavala A.M. (2022). Survey of Anesthesia, Sedation, and Non-Sedation Practices for Children Undergoing Repetitive Cranial or Craniospinal Radiotherapy. Cureus.

[34] Imbelloni L.E., Baroni D., Lara P.L.P., Valenccedil S., Pinho A.C., Calaccedil A.L., Rivoli A., de Morais Filho G.B. (2023). Prospective General Inhalation Anesthesia for Pediatric Patients Undergoing Radiotherapy. Pilot Project with 25 Children without Venous Access. Mathews J. Anesth..

[35] Ciccozzi A., Pizzi B., Vittori A., Piroli A., Marrocco G., Della Vecchia F., Cascella M., Petrucci E., Marinangeli F. (2022). The Perioperative Anesthetic Management of the Pediatric Patient with Special Needs: An Overview of Literature. Children.

[36] García-Aroca M.A., Fernández-de Miguel J.M., Franceschi M.A., Fernández-Vaquero M.A., Meléndez-Salinas D.A., Piñero-Merino M., Álvarez-Avello J.M. (2023). Inhalation Anesthesia without Any Intravenous Management for Pediatric Proton Beam Therapy. Paediatr. Anaesth..

[37] Punj J., Bhatnagar S., Saxena A., Mishra S., Kannan T.R., Panigrahi M., Pandey V. (2002). Propofol for Pediatric Radiotherapy. Indian J. Pediatr..

[38] Saied P., Afifi I., Mostafa I. (2021). Sedation and Anesthesia of Pediatric Patient for External Beam Radiotherapy (XRT). Benha Med. J..

[39] Christensen R.E., Nause-Osthoff R.C., Waldman J.C., Spratt D.E., Hearn J.W.D. (2019). Adverse Events in Radiation Oncology: A Case Series from Wake up Safe, the Pediatric Anesthesia Quality Improvement Initiative. Paediatr. Anaesth..

[40] Grebenik C.R., Ferguson C., White A. (1990). The Laryngeal Mask Airway in Pediatric Radiotherapy. Anesthesiology.

[41] Stackhouse C. (2013). The Use of General Anaesthesia in Paediatric Radiotherapy. Radiography.

[42] Sane S., Sinaei B., Golabi P., Talebi H., Rahmani N., Foruhar R., Kalashipour F., Gholamveisi B., Haki B.K. (2021). The Neurologic Complications Associated with Anesthesia in Pediatrics Treated with Radiotherapy Under Anesthesia. Iran. J. Pediatr..

[43] Oswald L., Al-Kadhimi S., Thorp N. (2025). Anaesthesia for Paediatric Radiotherapy: A Narrative Review. Anaesthesia.

[44] Verma V., Beethe A.B., LeRiger M., Kulkarni R.R., Zhang M., Lin C. (2016). Anesthesia Complications of Pediatric Radiation Therapy. Pract. Radiat. Oncol..

[45] Gutkin P.M., Skinner L., Jiang A., Donaldson S.S., Loo B.W., Oh J., Wang Y.P., von Eyben R., Snyder J., Bredfeldt J.S. (2023). Feasibility of the Audio-Visual Assisted Therapeutic Ambience in Radiotherapy (AVATAR) System for Anesthesia Avoidance in Pediatric Patients: A Multicenter Trial. Int. J. Radiat. Oncol. Biol. Phys..

[46] Wojcieszek E., Rembielak A., Bialas B., Wojcieszek A. (2010). Anaesthesia for Radiation Therapy—Gliwice Experience. Neoplasma.

